# Guillain-Barré Syndrome after Thrombolysis with Streptokinase

**DOI:** 10.4061/2010/315856

**Published:** 2010-12-26

**Authors:** Ertugrul Okuyan, Mehmet Akif Cakar, Mustafa H. Dinckal

**Affiliations:** ^1^Cardiology Clinic, Bağcılar Education and Research Hospital, Bagcilar, 34200 Istanbul, Turkey; ^2^Cardiology Clinic, Sakarya Education and Research Hospital, Sakarya, Turkey

## Abstract

Guillain-Barre syndrome (GBS) is an eponym for a heterogeneous group of immune-mediated peripheral neuropathies. We describe a case of GBS in a patient who recieved intravenous streptokinase therapy for acute anterior myocardial infarction. Clinical symptoms are thought to result from streptokinase-antibody complex mediated damage to the local blood-nerve barrier. Patient was treated with 5-days course of intravenous gammaglobulin and his outcome was good.

## 1. Introduction

Guillain-Barré syndrome (GBS) is an eponym for a heterogeneous group of immune-mediated peripheral neuropathies. A feature common in all GBS variants is a rapidly evolving polyradiculoneuropathy preceded by a triggering event, most often an infection [1]. Some cases of GBS after intravenous streptokinase administration have been reported previously [2–5]. We describe a new case of GBS in a patient who received intravenous streptokinase therapy for acute anterior myocardial infarction.

## 2. Case Report

A man 52-year-old taxi driver has been admitted to our clinic with a 1-hour onset chest pain. On electrocardiography, signs of hyperacute anterior wall myocardial infarction were evident. Streptokinase 1500000 unit was administrated over 1 hour intravenously. Reperfusion was positive clinically. Detailed medical history of the patient revealed no exposure to any drugs or toxins. Additionally, there was no evidence of any upper respiratory or gastrointestinal infection within the last 2 months. Medical history was also negative for arterial or venous embolism, connective tissue disease, and vasculitis. He was discharged on the 7th day with oral aspirin, metoprolol, spironolactone, simvastatin, and ramipril. Eleven days after discharge, he complained of progressive weakness on his legs and distal parasthesias. He was unable to walk without aid. On neurological examination, speech was dysarthric, left peripheral type facial paralysis, glove- and stocking-type sensorial impairment and absence of deep-tendon reflexes were prominent findings. Fundoscopic examination was normal. Cranial MRI revealed nothing. Blood chemistry was normal except hyperglycemia. Cerebrospinal fluid (CSF) analysis showed elevation of protein levels (216 mg/dl). CSF cultures were negative. Electromyographic investigation revealed extensive sensorymotor demyelinized type peripheral neuropathy, supporting the diagnosis of GBS. Patient was treated with 5-days course of intravenous gammaglobulin. Patient's outcome was good and 1 month later he was able to walk without aid, and 2 months later he was able to work.

## 3. Discussion

Streptokinase is a foreign protein derived from group C streptococci which might induce an immunological reaction leading to GBS [6]. It is important to remember that outside of certain institutions with interventional capability, fibronolytic therapy is still the most common form of acute-reperfusion therapy that is used for ST-elevation myocardial infarction. Therefore, streptokinase is still being used as a fibrinolytic therapy regimen for acute myocardial infarction worldwide, especially in developing countries and in some developed countries where facilities of immediate percutaneous coronary intervention is unavailable. Clinical symptoms are thought to result from streptokinase-antibody complex mediated damage to the local blood-nerve barrier [7]. GBS after acute myocardial infarction treated with reteplase also has been reported [8]. It is proposed that high-creatine kinase from significant muscle injury might be a possible immunological precipitant. 

In conclusion, GBS can be seen in the late course of acute myocardial infarction suggestive of triggered autoimmune mechanisms. After exclusion of more likely causes, diagnosis should be considered in patients who develop parasthesia and muscular weakness after 10–30 days of infarction, especially if thrombolysed with streptokinase. 
